# Emotional warmth and cyberbullying perpetration attitudes in college students: Mediation of trait gratitude and empathy

**DOI:** 10.1371/journal.pone.0235477

**Published:** 2020-07-14

**Authors:** Liang Chen, Yandong Wang, Hongze Yang, Xiaohua Sun

**Affiliations:** 1 School of Marxism, University of Science and Technology Liaoning, Anshan, Liaoning Province, China; 2 School of Business Administration, University of Science and Technology Liaoning, Anshan, Liaoning Province, China; 3 Organization Department of Party Committee, University of Science and Technology Liaoning, Anshan, Liaoning Province, China; University of La Rioja, SPAIN

## Abstract

Based on Social Learning Theory and the General Aggression Model, this study aims to explore the relationship between parental emotional warmth and the cyberbullying perpetration attitudes of college students and the mediating roles of trait gratitude and empathy. Using the stratified cluster random sampling method, 1198 college students (716 boys and 482 girls with an average age of 20.44 years) were tested using the subscale of the Parenting Styles Instrument, the Basic Empathy Scale, the Gratitude Questionnaire-6, and the Cyberbullying Attitude Questionnaire. Results: Emotional warmth, trait gratitude, cognitive empathy, and affective empathy all demonstrated significantly positive relationships with each other (*rs* from .175 to .403, *ps* < 0.01) and negative correlations with cyberbullying perpetration attitudes (*rs* from -.137 to -.306, *ps* < 0.01). Emotional warmth can exert an impact on cyberbullying perpetration attitudes through three fully mediating paths: the mediating roles of trait gratitude (41.91% of the total effect), cognitive empathy (14.5% of the total effect), and the chain mediating roles of trait gratitude–cognitive empathy (19.5% of the total effect). The results may have important implications for future studies to develop effective interventions for cyberbullying.

## 1 Introduction

Cyberbullying involves the use of information and communication technologies (ICTs) to carry out a series of acts as in the case of direct cyberbullying, or an act as in the case of indirect cyberbullying, intended to harm another person (the victim) who cannot easily defend him or herself [1]. Studies have found that the incidence rate of cyberbullying perpetration has reached 34.84% among senior high school students in China [2]. Similar results in a Turkish study showed a much higher prevalence, with 35.7% of secondary school students admitting perpetration [3]. Moreover, 12.1% of Belgian secondary school participants had cyberbullied others in the last three months before the study [4]. Based on the use of different measurements of cyberbullying perpetration, although it is difficult to compare prevalence rates between countries, research has shown that cyberbullying perpetration is a universal problem among adolescents [5]. Cyberbullying perpetration experienced by adolescents in their middle school years will continue to their college years, and cyberbullying perpetration strategies have a strong consistency [6]. Perpetrators of cyberbullying can cause serious harm to the mental and psychological status of cyberbullying victims because of cyberbullying features, such as anonymity, time-space cross, and rapid dissemination, resulting in low self-esteem, depression, anxiety, substance abuse, and even suicide [7]. Cyberbullying victimization during adolescence may continue until early adulthood [8]. Cyberbullying victims are likely to become new perpetrators to vent their emotions through cyberbullying [9], thus creating a vicious cycle. This study investigates the protective factors and mechanisms of college students’ cyberbullying perpetration attitudes. This helps to provide empirical evidence supporting studies on intervention against cyberbullying, considering that these attitudes have a significantly positive predictive effect on the occurrence and frequency of cyberbullying perpetration [10].

### 1.1 Emotional warmth and cyberbullying perpetration attitudes

According to Social Learning Theory (SLT), one can claim that impaired and aggressive social interactions with peers stem from conflictual and impaired family dynamics [11,12]. Howes (1987) asserts that the quality of the parent-infant relationship in early childhood is a critical etiological factor in the development of social skills [13]. Parenting style influences child development through its moderating influence on the relationship between parenting practices and developmental outcomes [14]. Previous studies have shown that maladapted social interactions and the escalation of child dysfunction result in more aversive clashes and aggression (in the family) that become a training environment for problematic relationships outside the home [15].

Bushman and Anderson (2002) proposed the General Aggression Model (GAM), also putting forward that individual aggression is the result of the combined effect of personal and situational factors [16]. Personal factors include gender, personality traits, attitudes, values, motives, beliefs, long-term goals, and any other consistent traits that the individual brings into the situation; situational factors mainly come from environmental stimuli around an individual [17]. Parenting styles have an important influence on the formation of individual aggression, especially in the family environment [18]. Different parents show various parenting styles. Positive parenting styles show emotional warmth, respect, and tolerance in raising and educating children, whereas, negative parenting styles exhibit autocracy, indifference, rejection, and overprotection during the process. Arrindell et al. (1999) divided parenting styles should be examined and divided into three types, namely, rejection, overprotection, and emotional warmth [19]. Rejection and overprotection are negative styles, whereas emotional warmth is a positive parenting style. Parenting warmth could result in higher social competence in children [20] and help them develop secure attachment [21]. Eiden et al. (2009) found that lower parental warmth would result in lower children’s self-regulation and externalizing behavior problems in the preschool years [22]. Findings used meta-analytic method revealed that the relationships between children’s self-reports about hostility/aggression and the perceived parenting warmth were negative [23]. Individuals who are affected by active parenting styles have few aggressive behaviors [24]. For example, emotionally warm parenting provides a safe raising and educating environment that helps children to effectively regulate negative emotions, thus reducing their aggressive behaviors [25,26].

Despite the limited available research, some studies have shown that positive parenting styles are inversely related to cyberbullying [27]. Studies have also found that an indulgent parenting style characterized by the use of reasoning and warmth can serve as a protective factor against children’s cyberbullying victimization [28]. Other studies also suggested that the authoritative parenting style, specifically, the support and warmth scale, correlated with less supportive cyberbullying attitudes and lower levels of cyberbullying in early adulthood [29,30]. Similar results were reported by Legate et al. [31] and Wang et al. [32], who found an opposite relationship between levels of parental support and involvement in cyberbullying perpetration.

### 1.2 Trait gratitude as a mediating variable

Gratitude refers to a positive emotion evoked by the perception that one has benefited from the generosity of another [33,34]. Gratitude can be categorized into trait gratitude and state gratitude.Trait gratitude has been found to an effective personality trait that promotes individuals’ adaptation to society [35] and mitigates against aggression [36]. Theoretical and empirical studies assert that gratitude is an ‘‘empathic emotion” [37] that prompts people to show sensitivity and concern toward others and to express reciprocity toward either the benefactor or uninvolved third parties [33,34,38,39]. The roots of gratitude is prosocial and empathic sensitivity to the feelings of others [40]. Individuals who possess traits for sensitivity to gratitude expressions feel more gratitude [34,39]. Therefore, people with higher levels of gratitude are more likely to have empathy.

According to SLT, a good parent–child relationship can promote the generation of gratitude in adolescents [41]. Various parenting styles have different predictive effects on gratitude. Among them, parental emotional warmth has a positive predictive effect on positive outcomes such as better interpersonal skills, since it includes praise and admiration as well as the expression of gratitude [42]. Further, adolescents who perceived more emotional warmth have reported higher scores of gratitude. In fact, mutual support is one of the foundational benefits of being in an intimate relationship. In those exchanges of support between family members, gratitude can easily appear in the context of ongoing parent-child relationships [40].

Gratitude increases mental well-being and mitigates against aggression. The GAM considers trait gratitude as one of the personal factors that can affect the generation of aggressive behaviors [36] and cyberbullying [17]. The theoretical understanding of the relationship between aggression and gratitude is based on a hypothesis that perceiving oneself to profit from the benefits and positive intentions of the benefactor inclines someone away from self-interest toward helpfulness and away from violence [36]. Gratitude is known to correlate with higher levels of empathy, and empathic concern for others may also promote prosocial motives and a buffer against violence. Evidence has been reported that gratitude correlates with reduced behavioral aggression in participants who are insulted and that empathy has a significant mediation effect on the relationship between gratitude and lower aggression [36]. However, previous studies did not make a further distinction between different functions of cognitive and affective empathy, as those two subscales of empathy are relatively independent, according to the self-reported measures and neuroscientific evidence [43,44]. Meanwhile, to our knowledge, the relationship between gratitude and cyberbullying has not been examined.

### 1.3 Empathy as a mediating variable

Empathy, as a fundamental human personality trait, refers to the psychological process in which a person puts herself or himself in others’ shoes, and he or she identifies and experiences other people’s emotions and feelings [45]. It is a psychological phenomenon that occurs in interpersonal interactions. Empathy can improve the incidence of pro-social behavior and effectively inhibit aggressive behavior [46]. Studies have divided empathy into cognitive and affective empathy [47,48]. Cognitive empathy focuses on conscious deliberation and judgment of people's emotions and putting these into perspective, and may support strategic social interactions and communication, whereas affective empathy mainly involves feeling and experiencing other people’s emotional state [49].

SLT holds that children’s empathic ability is acquired through the observation of speeches and postures expressing the emotions of the people in their surroundings [11]. Thus, positive parenting styles may affect the development of children’s empathy [50]. Understanding and emotional warmth can promote children’s emotional stability, optimism, and sincerity, thus helping them to adapt to their environment, improving their empathic ability [51].

GAM suggested that empathy, as an individual factor, was one of the predictor variables related to cyberbullying perpetration [49,52,53,54]. Schultze-Krumbholz and Scheithauer (2009) found that the empathy of cyberbullying perpetrators and victims is lower than that of individuals without any cyberbullying experience [55]. Although cognitive and affective empathy are all associated with cyberbullying perpetration, previous studies have found that lower cognitive empathy is a stronger predictor of cyberbullying perpetration than affective empathy is [49,53], as perpetrators cannot adequately experience the emotional states of their victims in the network environment. For example, in a study of Ang and Goh (2010), among participants with low affective empathy, both girls and boys with low level of cognitive empathy reported being involved in more cyberbullying perpetration than did those with high levels of cognitive empathy [52]. Among girls with high affective empathy, low and high levels of cognitive empathy resulted in similar levels of cyberbullying perpetration. Crick (1995) found that children who use indirect methods of bullying are not able to take the perspective of others, have low cognitive empathic responsiveness, which leads to increased levels of indirect bullying [56]. Inferring cognitive empathy might be an adequate means to combat and prevent cyberbullying perpetration attitudes [57].

Although the GAM presents protective factors for cyberbullying perpetration [17], the mechanism that leads to cyberbullying perpetration attitudes is unclear. From the above, we proposed a hypothetical model with emotional warmth as the independent variable, cyberbullying perpetration attitudes as the dependent variable, and trait gratitude, and empathy as the mediating variables. Therefore, three hypotheses were proposed:

H1. Emotional warmth has a significant negative relationships with cyberbullying perpetration attitudes.
H2. Trait gratitude and cognitive empathy play mediating roles in emotional warmth and cyberbullying perpetration attitudes.
H3. Trait gratitude and cognitive empathy plays a chaining mediating role in emotional warmth and cyberbullying perpetration attitudes.

## 2 Method

### 2.1 Ethics statement

The study protocol was approved by the Research Ethics Committee of the University of Science and Technology Liaoning (China).

### 2.2 Participants

A stratified cluster random sampling method was employed to select 1,198 freshman to senior-year college students from three universities in China as our research participants. A total of 1,300 questionnaires were distributed during the students' self-study time. After excluding invalid questionnaires using inclusion and exclusion criteria (52 questionnaires with the same responses to all questions, 47 questionnaires missing more than half of the data), 1,198 valid questionnaires were collected without three extreme outliers, with an effective recovery rate of 92.15%. The number of students from freshman to senior year was 297 (24.9%), 329 (27.4%), 295 (24.6%), 277 (23.1%), respectively. Of these, 657 students (54.9% of the participants) were majoring in engineering, 215 (17.9%) were majoring in science, and 326 (27.2%) were majoring in liberal arts. The participants included 716 boys (59.8%) and 482 girls (40.2%). In terms of residence, 558 (46.6%) had an urban residence registration and 638 (53.3%) had a rural residence registration. The ages of the participants ranged from 17 years to 23 years, with an average age of 20.44 years and a standard deviation of 1.45.

### 2.3 Measures

#### 2.3.1 Parenting styles instrument

The simplified Chinese version of the parenting styles instrument, revised by Jiang et al. [58], was adopted in this study. Each of the father and mother versions of the questionnaire had 21 questions with the same content, including three dimensions, namely, emotional warmth (eight items), overprotection (six items), and rejection (seven items). A four-point scoring program was used, where 1 represented “never” and 4 represented “always.” The average score was calculated. The higher the score was, the higher the level of the perceived parenting styles would be. In this study, the subscale of emotional warmth was used for the analysis (e.g., Mother/Father praised me). The mean of the gross scores of the father and mother version subscales was determined to calculate the reliability value. The goodness-of-fit indexes of correction two factors were *χ*^*2*^ = 355.399***, *df* = 96, CFI = 0.973, TLI = 0.966, SRMR = 0.041, RMSEA = 0.049.

#### 2.3.2 Basic Empathy Scale

Basic Empathy Scale (BES), compiled by Darrick et al. in 2006, is a self-assessing questionnaire for empathy [59]. Its items are generated on the basis of the definitions of affective and cognitive empathy and drawn from four basic emotions (fear, sadness, anger, and happiness), avoiding the impact of social desirability. The revised Chinese version of the BES consisted of 20 items, the affective empathy scale contained 11 items (e.g., I get caught up in other people’s feeling easily), and the cognitive empathy scale comprised 9 items (e.g., I have trouble figuring out when my friends are happy). The participants were assessed on a scale of 1 to 5 based on their own level, where 1 meant “completely disagree,” and 5 meant “completely agree.” The scale contained two subscales, namely, affective empathy and cognitive empathy. The goodness-of-fit indexes of correction two factors with methodological effect were *χ*^*2*^ = 346.325***, *df* = 129, CFI = 0.933, TLI = 0.901, SRMR = 0.030, RMSEA = 0.037.

#### 2.3.3 Gratitude Questionnaire-6

Six items (e.g., I am grateful to a wide variety of people), with Likert seven-point scoring, was adopted, from “totally disagree” to “completely agree” scored from 1 to 7 points, respectively, using the Gratitude Questionnaire designed by McCullough et al. [33] and revised by Li et al. [60]. The average score of the six items were calculated. The higher the score was, the stronger the individual’s trait gratitude would be. The goodness-of-fit indexes in CFA were *χ*^*2*^ = 134.584***, *df* = 9, CFI = 0.944, TLI = 0.906, SRMR = 0.036, RMSEA = 0.109.

#### 2.3.4 Cyberbullying Attitudes Measure (CAM)

Barlett, Helmstetter, and Gentile’s (2016) CAM among college students was used [61]. The translation and adaptation procedures for this scale were performed following the guidelines developed by the International Test Commission. Considering the advantages and disadvantages of the forward-adaptation and backward-adaptation methods, all items of the original instrument were translated into Chinese by bilingual translators. The Chinese versions were then translated back into English by a psychology professor who was proficient in both languages. Then, the final Chinese and the English back-translated versions were compared by another psychology professor and six psychology graduate students. Several wording revisions were done to ensure consistency with the Chinese culture.

The Chinese version of the CAM contained 10 items using Likert’s five-point scoring, where 1 meant “completely disagree,” and 5 meant “completely agree.” The higher the score was, the stronger the individual’s cyberbullying perpetration attitudes would be. The questionnaire included two subscales, namely harmful cyberbullying perpetration attitudes (e.g., Teasing or making fun of others with harmful comments online is fun to me) and general cyberbullying perpetration attitudes (e.g., Sending mean electronic messages to others is less harmful than face-to face communication). Each of these subscales contained five items. The goodness-of-fit indexes of correction two factors were *χ*^*2*^ = 134.584***, *df* = 9, CFI = 0.931, TLI = 0.906, SRMR = 0.048, RMSEA = 0.094.

### 2.4 Procedure

The researcher started by reading the instructions and informing the respondents about the anonymity and confidentiality of the research. Then, participants signed an informed consent form and completed a few questionnaires including the measures in this study for emotional warmth, trait gratitude, empathy, and cyberbullying perpetration attitudes in classrooms. The researcher answered questions at all times. The time for completing the questionnaire was about half an hour. At the end of the research, the participants submitted the completed questionnaires to the researcher.

### 2.5 Data analysis

The percentage of missing values was calculated in each subscale (see Table 1). The series mean method in SPSS was used to deal with missing values. The series mean refers to replacing missing values with the mean for the entire series. SPSS 23.0 (IBM Corporation, New York, USA) was used for descriptive statistics, reliability analysis, correlation analysis, *t*-test, one-way ANOVA, and exploratory factor analysis. Mplus 8.1 [62] was employed to perform mediation analyses of the structural equation model (SEM) and Item Parceling of confirmatory factor analysis (CFA). In the first prerequisite step, the total effect of emotional warmth on cyberbullying perpetration attitudes was examined. In the second step, the mediation model was established to check the significance of each of the path coefficients. In the third step, the confidence interval of path coefficients was estimated [63]. Several goodness-of-fit indices were used, including the chi-squared goodness-of-fit statistic, the Tucker–Lewis Index (TLI), the comparative fit index (CFI), the root mean square error of approximation (RMSEA), and the standardized root mean square residual (SRMR). TLI and CFI values less than 0.90 imply an acceptable fit; a value of 0.08 or lower for RMSEA and SRMR indicates a moderate fit, while values higher than 0.08 signify a poor fit [64–68].

**Table 1:** Correlation matrices, gender and age differences in key variables (*N* = 1198).

Variables	*M*	*SD*	% of Missing	McDonald’s Omega	Trait gratitude	Cognitive empathy	Affective empathy	CAP	*t* (Cohen's d)	*F* (*η*^2^_p_)
Emotional warmth	2.878	.545	.31	.891	.403**	.277**	.175**	-.205**	-2.21* (.130)	2.94** (.015)
Trait gratitude	5.910	.835	.07	.826	-	.366**	.234**	-.306**	-2.519* (.151)	1.962 (.010)
Cognitive empathy	3.717	.475	.23	.730		-	.273**	-.266**	-2.115* (.126)	3.663** (.018)
Affective empathy	3.490	.532	.24	.750			-	-.137**	-7.781*** (.463)	.923 (.005)
CAP	1.864	.656	.11	.884				-	7.058*** (.421)	.663 (.003)

*p < 0.05

**p < 0.01, CPA: cyberbullying perpetration attitudes.

## 3 Results

### 3.1 Common method bias test

All questionnaires in this study were completed anonymously to improve the authenticity of the individuals’ responses. The 78 items of the four scales were subjected to exploratory factor analysis with Harman’s single factor test. A total of 22 factors with characteristic roots greater than 1 were extracted, and the explanatory power of the first factor was only 13.791%, which was lower than the judging criterion of 40%. Therefore, common method bias did not exist using this method.

### 3.2 Descriptive statistics and correlation analysis

Descriptive statistics and correlation analysis were carried out on emotional warmth, trait gratitude, cognitive empathy, affective empathy, and cyberbullying perpetration attitudes. McDonald's omega coefficients for measurements were listed in Table 1. Correlation analysis results (see Table 1) showed that emotional warmth, trait gratitude, cognitive empathy, and affective empathy all demonstrated significantly positive relationships with each other. Cyberbullying perpetration attitudes significantly negative correlated with the variables mentioned above.

### 3.3 Gender and age differences

We tested for gender differences and the result showed that males and females significantly differed in the scores of emotional warmth, trait gratitude, cognitive empathy, affective empathy, and cyberbullying perpetration attitudes (see Table 1). One-way ANOVA was carried out to analyze the variables’ mean comparisons between different age groups. The findings revealed significant main effects of emotional warmth and cognitive empathy (see Table 1). Therefore, gender and age could be tentative covariates in SEM.

### 3.4 Analysis of mediating effects

This study packaged the items of cognitive and affective empathy subscales according to the Item Parceling to simplify the measurement structure of the mediation model. First, single-dimensional confirmatory factor analysis was performed on each of the subscale items [69]. Then, the items were packaged on the basis of the factor load size of each item, thus maintaining the total value balance of the factor load in each of the item parcels [70]. Therefore, items in the cognitive and affective empathy subscales were respectively packaged into three item parcels as observational variables. In addition to trait gratitude (items as observational variables), each dimension was taken as an observational variable for emotional warmth and cyberbullying perpetration attitudes.

SEM with a bootstrapping and Markov chain Monte Carlo (MCMC) method was used to test the mediating effects of trait gratitude, cognitive empathy, and affective empathy, controlling for gender and age as covariates. First, the significance of the total effect *c* was examined. The total effect of emotional warmth on cyberbullying perpetration attitudes was −.241 (c1), with a significant total effect path coefficient (*p* < .001), and all fitting indices of the total effect model were good (*χ*^*2*^ = 3.99***, *df* = 5, CFI = 1.000, TLI = 1.000, SRMR = .006, and RMSEA = .000).

**Fig 1:**
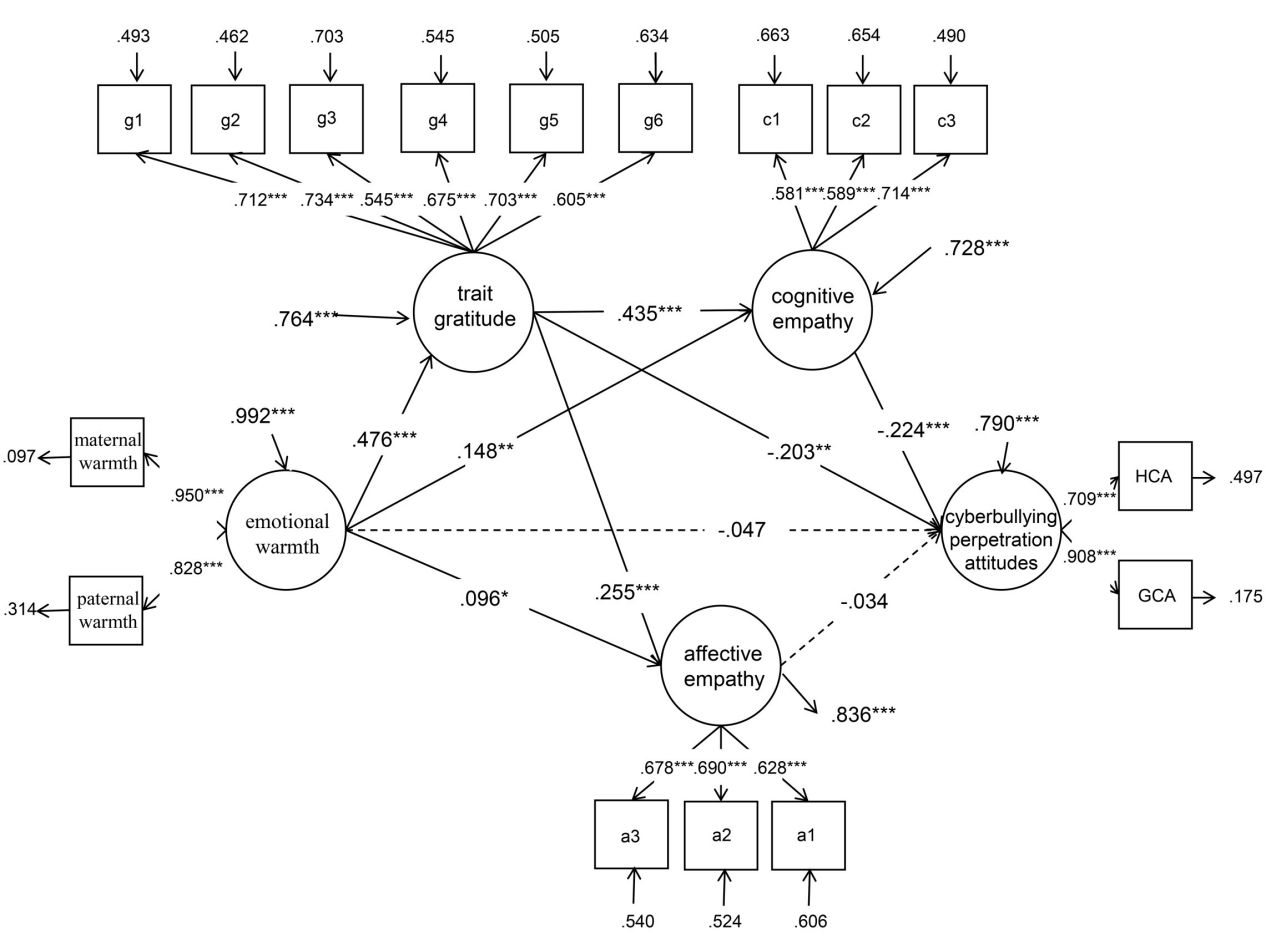
Trait gratitude and empathy as the mediating variables between emotional warmth and cyberbullying perpetration attitudes. *p < 0.05, **p < 0.01, ***p < 0.001.

**Fig 2:**
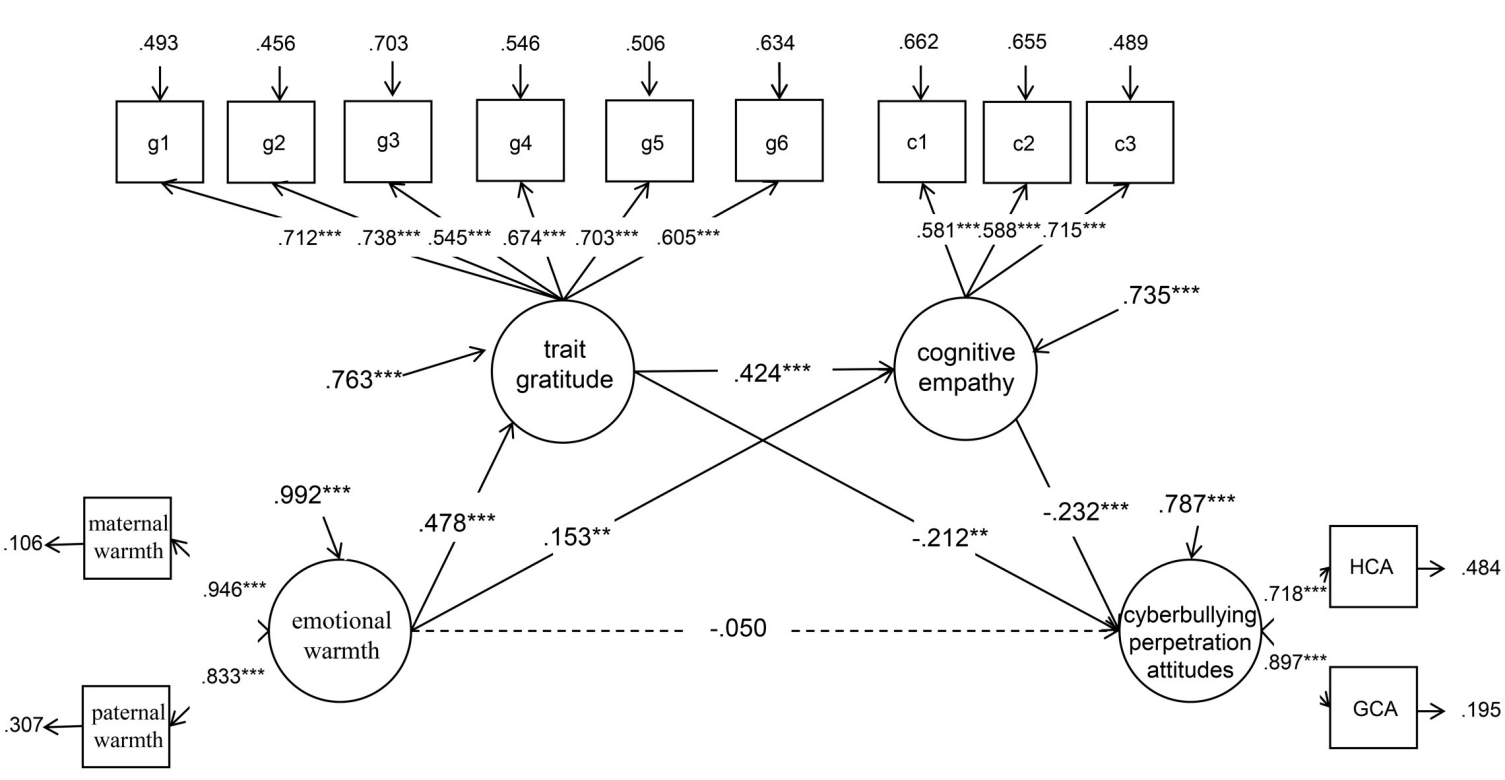
Trait gratitude and cognitive empathy as the mediating variables between emotional warmth and cyberbullying perpetration attitudes. **p < 0.01, ***p < 0.001.

Second, the significance of each of the path coefficients was checked in turn. In this study, Model A was constructed (Fig 1). The mediation model was built with emotional warmth as the independent variable; cyberbullying perpetration attitudes as the dependent variable; and trait gratitude, cognitive empathy, affective empathy as the mediating variables. The fitting indices of the model were good (*χ*^*2*^ = 585.048***, *df* = 117, CFI = .926, TLI = .904, SRMR = .044, and RMSEA = .058). Structural equation analysis for model A showed that the path coefficient of affective empathy related to cyberbullying perpetration attitudes was not significant (*p* > 0.05). Then, we deleted affective empathy to establish Model B (Fig 2). The fitting indices of the Model B were good (*χ*^*2*^ = 392.187***, *df* = 77, CFI = .942, TLI = .921, SRMR = .034, and RMSEA = .058). All path coefficients reached the significant level, and the normalized factor loadings of the observational variables were significant, indicating that the model was standard. Therefore, trait gratitude and cognitive empathy played a fully mediating role in the influence of emotional warmth on cyberbullying perpetration attitudes.

Last, the confidence interval of path coefficients was estimated, and 1,000 samples were randomly sampled. The results showed that the bias-corrected bootstrap 95% confidence interval for the total indirect effects of trait gratitude and cognitive empathy in Model B did not contain 0 (−.385, −.085), indicating that the two mediating variables had significant mediating effects between emotional warmth and cyberbullying perpetration attitudes (.272, 75.91% of the total effect). The mediating effect included three indirect effects (see Table 2). First was indirect effect 1 caused by the path of emotional warmth influencing cyberbullying perpetration attitudes through trait gratitude, with a confidence interval containing no 0 (−.190, −.052), indicating that the mediating effect of trait gratitude between emotional warmth and cyberbullying perpetration attitudes was significant (.101, 41.91% of the total effect). Second was indirect effect 2 caused by the path of emotional warmth related to cyberbullying perpetration attitudes through cognitive empathy, with a confidence interval containing no 0 (−.081, −.015), indicating that the mediating effect generated by this path (.035, 14.5% of the total effect) was significant. Third was indirect effect 3 caused by the path of emotional warmth related to cyberbullying perpetration attitudes through trait gratitude and cognitive empathy, with a confidence interval containing no 0 (−.088, −.032), indicating that the mediating effect of this path (.047, 19.5% of the total effect) was significant.

**Table 2:** Bootstrap analysis of mediating effect test.

Influence path	Standardized indirect effect estimation	95% confidence interval	
		lower limit	upper limit
EW→trait gratitude→CPA	∣.478 × (-.212)∣ = .101	-.190	-.052
EW→cognitive empathy→CPA	∣.153 × (-.232)∣ = .035	-.081	-.015
EW→trait gratitude→cognitive empathy→CPA	∣.478× .424 × (-.232)∣ = .047	-.088	-.032

EW: emotional warmth, CPA: cyberbullying perpetration attitudes.

## 4 Discussion

The present study aimed to assess an integrated theory-driven process-model of cyberbullying perpetration attitudes incorporating environmental traits (emotional warmth) and individual traits (trait gratitude and empathy) related to cyberbullying perpetration attitudes.

### 4.1 Direct effects of emotional warmth on cyberbullying perpetration attitudes

The results of this study showed that emotional warmth had a significantly negative predictive effect on the cyberbullying perpetration attitudes of college students, thus supporting H1. This findings from this study is in contrast with previous studies [29,30]. Perceived emotional warmth played an important role in the formation and development of individuals’ positive psychological traits and served as a protective factor in negative psychology and problem behaviors [23]. Emotional warmth can promote the development of individuals’ social ability [22], affecting peer attachment in their adolescent years through which an internal working model can be built to influence the interpersonal communication among individuals [21]. According to SLT [11], parenting styles that are full of warm emotions can set an example of interpersonal interaction for children to help them build and maintain positive peer relationships through imitation. Therefore, individuals tend to resolve contradictions and conflicts in cyber social communication in positive ways, thus reducing cyberbullying perpetration attitudes.

### 4.2 Mediating effects of trait gratitude and cognitive empathy

Our results supported H2. Trait gratitude plays a fully mediating role between emotional warmth and college students’ cyberbullying perpetration attitudes, with an effect size of 36.99%. Our study found that emotional warmth had a positive predictive effect on trait gratitude. The study also revealed that trait gratitude could negatively predict college students’ cyberbullying perpetration attitudes. Adolescents with a high degree of trait gratitude also have a high degree of pro-social motivation and behavior, thus becoming less prone to situational influences for cyberbullying behavior [36]. This argument further extends our understanding of trait gratitude by suggesting that this mechanism is likely to underlie a more general personality trait-formation process that mitigates the enactment of cyberbullying perpetration. Therefore, when an individual has a high degree of gratitude, he or she finds it difficulty to develop a cyberbullying perpetration attitudes.

The results of this study suggested that cognitive empathy played a fully mediating role between emotional warmth and college students’ cyberbullying perpetration attitudes, with the effect sizes of 13%. Emotional warmth has a significant influence on the development of empathy. Empathy is a socialized emotion because the family environment is the first site of a child’s socialization and parents are the first teachers before a child steps into society [14]. Parenting styles remarkably influence children’s character formation and personality development. Emotional warmth is a positive parenting style and a positive response to children’s psychological needs, thus promoting their pro-social ability. Children brought up in such an environment show empathy toward others’ emotions and have a strong empathic ability. Although this study found a negative correlation between affective empathy and cyberbullying perpetration attitudes, affective empathy has no mediating effect. Affective empathy needs more direct contacts to be expressed than cognitive empathy does. The conditions for affective empathy may be suppressed in cyberspace. Therefore, the mediation analysis only indicated that cognitive empathy plays a significant mediating role in the relationship between parental emotional warmth and college students’ cyberbullying perpetration attitudes.

Our results also supported H3, which suggested that trait gratitude–cognitive empathy played a significant mediating role between emotional warmth and college students’ cyberbullying perpetration attitudes, with effect sizes of 17.89%. The results of this study showed that trait gratitude and cognitive empathy have a significantly negative predictive effect on college students’ cyberbullying perpetration attitudes, and both have an inhibitory effect on their cyberbullying perpetration attitudes. The significantly positive correlation between trait gratitude and cognitive empathy indicates that a college student with strong trait gratitude has a high degree of cognitive empathy. This study found that trait gratitude can inhibit cyberbullying perpetration attitudes of college students through cognitive empathy.

### 4.3 Research shortcomings and recommendations for future research

This study had several shortcomings. First, the study adopted the self-reported questionnaire method. Systematic errors exist, although the study adopted a common method bias test to improve the validity of the results. Other research methods, such as other-report questionnaire and laboratory methods, can be used to verify our results. Second, a cross-sectional study was conducted to examine the influence of emotional warmth on cyberbullying perpetration attitudes. The participants filled in their perceived parenting styles information with their memory; therefore, memory bias might exist, that influences the authenticity of the results. Meanwhile, considering the difficulties of examining mediation mechanisms in cross-sectional studies, longitudinal studies may be adopted to improve the research validity in the future. Third, gender and age were included as covariates in SEM; however, other covariates should be analyzed in future studies, such as IQ, school performance, etc. Fourth, this study only addressed cyberbullying perpetration attitudes, not the actual behavior. In consideration of the general weak association between cyberbullying perpetration attitudes and the actual behavior [71,72], the relationship between attitudes expressed and cyberbullying perpetration behavior should be tested in future studies. Fifth, some of the correlations among this study’s variables could be explained by the fact that almost all of them (maybe with the exception of affective empathy) are probably accounting for the cognitive component of the specific construct measured. Cluster analysis or exploratory factor analysis could be considered to verify this hypothesis. Last, this study chose participants from only one college, which is a university of science and engineering. In the future, samples of undergraduate and junior college students in the arts and normal education can be chosen to expand the representativeness of the samples.
